# The Relationship between miR-5682 and Nutritional Status of Radiotherapy-Treated Male Laryngeal Cancer Patients

**DOI:** 10.3390/genes15050556

**Published:** 2024-04-27

**Authors:** Marcin Mazurek, Anna Brzozowska, Mirosław Maziarz, Teresa Małecka-Massalska, Tomasz Powrózek

**Affiliations:** 1 Department of Human Physiology of Chair of Preclinical Sciences, Medical University of Lublin, 20-059 Lublin, Poland; miroslawjanmaziarz@gmail.com (M.M.); teresa.malecka-massalska@umlub.pl (T.M.-M.); tomaszpowrozek@gmail.com (T.P.); 2 Department of Radiotherapy, St. John of Dukla Lublin Region Cancer Center, 20-090 Lublin, Poland; annabrzo@poczta.onet.pl

**Keywords:** cancer cachexia, malnutrition, radiotherapy, head and neck cancer, miRNA

## Abstract

Background: Nutritional deficiencies are frequently observed in patients with head and neck cancer (HNC) undergoing radiation therapy. microRNAs (miRNAs) were found to play an important role in the development of metabolic disorders throughout regulation of genes involved in inflammatory responses. This study aimed to explore the correlation between pre-treatment miR-5682 expression and parameters reflecting nutritional deficits in laryngeal cancer (LC) patients subjected to radiotherapy (RT). Methods: Expression of miR-5682 was analyzed in plasma samples of 56 male LC individuals. Nutritional status of LC patients was assessed using anthropometric and laboratory parameters, bioelectrical impedance analysis (BIA) and clinical questionnaires. Results: A high expression of miR-5682 was associated with significantly lower values of BMI, fat mass, fat-free mass and plasma albumin at selected periods of RT course. miR-5682 allowed us to distinguish between patients classified with both SGA-C and low albumin level from other LC patients with 100% sensitivity and 69.6% specificity (AUC = 0.820; *p* < 0.0001). Higher expression of studied miRNA was significantly associated with shorter median overall survival (OS) in LC patients (HR = 2.26; *p* = 0.008). Conclusions: analysis of miR-5682 expression demonstrates a potential clinical utility in selection of LC patients suffering from nutritional deficiencies developing as a consequence of RT-based therapy.

## 1. Introduction

Malnutrition is a complex condition characterized by parallel loss of body weight and muscle mass (with or without corresponding adipose tissue loss), and its severe stage manifesting as cachexia cannot be completely reversed by normal dietary therapy [1,2]. Cancer malnutrition is defined as an imbalance between protein and energy turnover resulting from decreased food intake and enhanced catabolic condition caused by a tumor [3]. Malnutrition is defined by a weight loss of at least 5% in last 6 months with corresponding depletion of muscle function and systemic inflammatory response [4]. Additionally, malnutrition or cachexia refers to 70% of all cancer patients, and accounts for up to 22% of cancer mortality [1]. Head and neck cancer (HNC) is the sixth most commonly diagnosed neoplasm worldwide, and more than 90% of all HNCs represent squamous cell histology. HNC include cancers most frequently locating in the region of larynx (30–40%) [5,6]. Approximately 60% of cases are locally advanced or metastatic laryngeal cancer (LC) (stage III or IV) at the time of diagnosis [7]. Malnutrition in LC patients is frequently caused by the both difficulties in food intake and swallowing, due to the anatomic location of the tumor. Appetite loss and alterations regarding taste and smell senses are also important factors predisposing in this group of patients to the development of malnutrition [4]. Conversely, in 20.2–32.2% of therapy naive LC patients, cancer-related malnutrition or cachexia is diagnosed [2,8]. Critical weight loss (CWL), defined either as an unintentional loss of ≥5% of body weight in a one month or ≥ 10% within last six months, occurs in 20–50% of LC cases, and negatively affects patients’ survival [9,10]. Moreover, the percentage of patients with CWL may increase to 72–88% as a consequence of therapy, mainly of radiation therapy [10,11].

The immune system seems to play a leading role in the development of cancer-related malnutrition and cachexia by the overproduction of various inflammatory mediators (e.g., TNF-α, IL-1β, IL-6, INFγ). The concentration of pro-inflammatory cytokines gradually increases during the cancer course, and can be also intensified by applied therapy, mainly radiotherapy (RT) [12]. Specifically, RT-caused toxicity and therapy complications are commonly observed in malnourished LC patients, and affect either development of malnutrition or accelerate its progression toward severe malnutrition or even cause cachexia [11]. Malnutrition can be observed in 44–88% of HNC patients who received RT or chemoradiotherapy (C-RT) [13]. The negative impact of malnutrition on patients’ clinical picture and therapy outcomes encourages us to investigate biomarkers that could allow for early detection of malnutrition at its initial stage, and then the selection of patients at nutritional risk. Short (approximately 22 nucleotides in length), non-coding RNAs known as microRNAs (miRNAs) involved in the post-translational control of genes participating in the regulation of inflammatory response, metabolic conditions and therapy response are the promising candidate biomarkers reflecting malnutrition in HNC [14]. Several miRNAs, including miR-3184-3p, miR-423-3p, miR-1296-5p, miR-345-5p, miR-199a-3p, miR-423-5p, miR-532-5p and let-7d-3p were identified as regulators of myogenesis, muscle metabolism, and inflammatory course in cancer cachexia [15]. According to the bioinformatics (miRPath v4.0), miR-5682 is involved in the regulation of expression of genes involved in protein digestion and absorption process (KEGG term: hsa04974). By downregulation of *ESL* gene and *COL* gene family, the studied miRNA can lead to decrease in absorption of dietary proteins and, thus, participating in protein depletion (e.g., in the muscle tissue) under the malnutrition process. Additionally, for Wikipathays analysis, the top enrichment terms are as follows: factors and pathways affecting insulin-like growth factor (IGF1)-Akt signaling, PI3K-Akt signaling pathway, and Inflammatory response pathway. Based on the other bioinformatics tool (using TargetScan Human 7.2), it can be postulated that miR-5682 could potentially participate in the regulation of the following genes regulating inflammatory response: *TNFRSF1A*, *TNFRSF11A*, *TNFSF18*, *TNFRSF9*, *IL1RAP*, *IL1B*, *IL6ST*, *IRAK3*, *NFKBIA*, *TLR4*. In the present study, we investigated the relationship between expression ofmiR-5682 and presence of nutritional disorders in patients subjected to RT-based therapy due to advanced LC. The secondary objective was to evaluate the prognostic role of miR-5682 in this group of patients.

## 2. Material and Methods

### 2.1. Study Group

The study group included 56 surgically treated male LC patients (mean age: 66 years) with a locally advanced or advanced stage of the disease. All study participants were treated by an RT-based therapy regimen between 2014 to 2017 at the Department of Oncology of the Medical University of Lublin. The specific inclusion criteria for the study were defined as follows: age > 18 years, male gender, histopathologically confirmed cancer in the larynx, locally advanced or advanced stage of the disease according to the TNM classification (8th edition: III–IV), treatment protocol based on intensity-modulated radiotherapy (IMRT) technique, with or without sequential and/or simultaneous chemotherapy, and receiving the complete radiation dose (7 weeks of treatment). The exclusion criteria were as follows: autoimmune diseases, presence of any concomitant cancer or previous cancer, poor performance status, withdrawal from RT due to severe toxicity. The International Statistical Classification of Diseases and Related Health Problems (ICD) scale for assessment of alcohol consumption was used. The patient’s performance status (PS) was evaluated according to the Eastern Cooperative Oncology Group (ECOG-WHO) scale. The study protocol was approved by the Bioethical Commission at the Medical University of Lublin (no of consent: KE-0254/64/2017). Before the enrolment to the study group, all patients signed informed consent. Detailed characteristics of study group are presented in Table 1.

### 2.2. Treatment Protocol

All of the patients were treated with the IMRT-based technique using the ONCOR (Siemens) linear accelerator. A total of 54–70 Gy (~2 Gy daily) doses were administrated. The group with advanced cancer received 35 fractions of irradiation on tumors and enlarged lymph nodes (total 70 Gy). Post-surgical patients with volume risk received 33 fractions of irradiation for a total dosage of 66 Gy. The groups with an average and low risk received irradiation doses of 60 and 54 Gy, respectively. Elective lymph nodes were treated with either 54 or 60 Gy. Selected patients received cisplatin and 5-fluorouracil, in addition to IMRT in 1–4 cycles of chemotherapy (C-RT). The entire therapy regimen lasts 7 weeks in all scheduled patients.

### 2.3. Nutritional Status Assessment

The nutritional status of the patients was evaluated using anthropometric parameters (body mass, body mass index (BMI)), laboratory testing, and bioelectrical impedance analysis (BIA) for each patient on the same day (from 24 to 48 h before the start of RT). Moreover, according to the SGA questionnaire, participants were divided into three groups: well-nourished (SGA-A), moderately malnourished (SGA-B), and severely malnourished (SGA-C). Furthermore, the prediction risk of malnutrition was assessed using the NRS-2002. Nutritional risk index (NRI) was calculated using the following formula: NRI = [1.519 × serum albumin (g/L)] + 41.7 × (current weight/usual weight) in order to assess malnutrition risk. According to the NRI score, patients were divided into four groups: no risk (>100), mild risk (97.5–100), moderate risk (83.5–97.5), and severe risk (<83.5) of malnutrition. A weight loss of >5% since the start of RT to week IV or >6.25% to week VII was defined as critical weight loss (CWL). During laboratory examination, the serum concentration of albumin, prealbumin, total protein (TP), transferrin, and C-reactive protein (CRP) was analyzed. Anthropometrical parameters included body mass index (BMI), body weight, and weight loss during the RT course.

### 2.4. Bioelectrical Impendence Analysis

Body composition parameters were obtained from BIA before, during and after the commencement of RT (week I, IV and VII). ImpediMed bioimpedance analysis SFB7 BioImp v1.55 device (Pinkenba, QLD, Australia) was used for BIA. The following parameters reflecting patients’ nutritional status were obtained from BIA: fat-free mass (FFM) and fat mass (FM). Fat-free mass index (FFMI) and the normalized fat-free mass index (nFFMI) were calculated using the following formula: FFMI [kg/m^2^] = FFM [kg]/(height [m])^2^; nFFMI [kg/m^2^] = FFMI [kg/m^2^] + 6.1 × (1.8 − height [m]).

### 2.5. miRNA Expression Analysis

A total of 5 mL of whole blood was collected from all patients before the initiation of RT. Then, plasma samples were collected and stored at −80 °C until the miRNA isolation. miRNeasy Serum/Plasma Kit (Qiagen, Hilden, Germany) was used for miRNA purification from 200 μL of plasma according to the manufacturer’s protocol. Purified miRNA were reverse transcribed into complementary DNA (cDNA) with a dedicated kit (TaqMan Advanced miRNA cDNA Synthesis Kit, Thermo Fisher Scientific, Waltham, MA, USA). The amplification of targeted sequences was performed on a StepOnePlus device (Applied Biosystems, Foster City, CA, USA) using TaqMan Fast Advanced Master Mix (Thermo Fisher Scientific, Waltham, MA, USA) and TaqMan Advanced miRNA Assay probes (Assay name: hsa-miR-5682; Assay ID: 480144_mir; Thermo Fisher Scientific, Waltham, MA, USA) (Figure S1). Each sample was analyzed in triplicate. The expression level of miR-5682 was normalized on miR-26a-3p (internal reaction control) using 2^−ΔΔCt^ and 2^−ΔCt^ formulas.

### 2.6. Statistical Analysis

MedCalc v.15.8 computer software (MedCalc Software, Oostende, Belgium) was used in order to perform statistical analysis and generate figures. Results demonstrating *p* < 0.05 were considered statistically significant. Comparisons of the expression of the studied miRNA depending on clinical-demographic factors and nutritional status were analyzed by the U Mann–Whitney or the Kruskal–Wallis tests. Correlation between the miR-5682 expression, clinical–demographic factors, and nutritional status of patients were performed with Spearman’s rank correlation. Receiver operating curves (ROC) were used to determine the cut-off points and evaluate the diagnostic accuracy of miR-5682 in the detection of nutritional disorders in the studied patients. Kaplan–Meier estimator and Cox logistic regression models (with the calculation of the hazard ratio (HR) with 95% CI) were applied to assess factors affecting patients’ survival.

## 3. Results

### 3.1. Nutritional Assessment

Before RT, among the studied individuals, patients with moderate (SGA-B; 39.3%) and severe (SGA-C; 46.4%) malnutrition according to SGA scale predominated, and 39.3% of patients demonstrated CWL. In turn, after completion of the treatment (week VII), 18 patients with moderate (SGA-B; 32.1%) and 38 patients with severe malnutrition (SGA-C; 67.9%) were reported based on the SGA evaluation. We recorded that, of eight patients who were diagnosed as well-nourished (SGA-A) before RT, all of them were then found malnourished. Two of them were diagnosed moderately malnourished (SGA-B; 25%), whereas six others were severely malnourished (SGA-C; 75%) after the completion of treatment. Moreover, 6 out of 22 patients (27.3%) classified to SGA-B group prior to treatment were qualified to SGA-C after the completion of RT. In addition, 75% of patients had moderate and 10.7% had severe risk of malnutrition, as evidenced by NRI assessment. On the other hand, only 28.6% of patients had higher risk of nutritional disorders (defined as NRS > 3) according to NRS-2002. Severely malnourished (SGA-C) patients had significantly lower plasma albumin concentration measured at week I compared to moderately (SGA-B) and well-nourished (SGA-A) patients (median: 3.22 vs. 3.31 vs. 3.82; *p* = 0.0001, respectively). After completion of the treatment, we observed non-significantly higher albumin concentrations in the SGA-C group compared to moderately malnourished patients. After the RT course, patients in SGA-C group had insignificantly higher plasma albumin concentration, in contrast to SGA-B individuals (median: 3.11 vs. 3.21; *p* = 0.293). We also recorded that, prior to the RT, plasma albumin concentration was within the normal range in 44/56 (78.6%) patients; however, after the completion of RT, this percentage decreased to 20/56 (35.7%); *p* < 0.001. Detailed nutritional characteristics of patients are summarized in Table 2.

### 3.2. Relationship between Expression of miR-5682 and Nutritional Status of LC Patients

We examined the relationship between the studied miRNA levels and the clinical and demographic factors of LC male patients. We only noticed that patients with poorer performance status according to the ECOG-WHO (>1) had significantly higher expression of miR-5682 compared to the others (median: 1.08 vs. 5.94; *p* = 0.015) (Table S1).

In order to compare the nutritional status of LC patients at different RT- measurement points according to the miR-5682 expression, patients were divided into the two following groups, depending on median miRNA expression (median: 1.29) measured prior to RT: with either low (<1.29) or high (≥1.29) miRNA expression. In our study 28 patients had low and 28 had high expression of studied miRNA (Table 3). Individuals who had low miR-5682 expression level presented significantly higher BMI before the commencement of RT (week I) (median: 23.78 vs. 21.96 kg/m^2^; *p* = 0.018), at the fourth week (IV) of treatment (median: 22.67 vs. 19.82 kg/m^2^; *p* = 0.009), and after the completion of therapy (week VII) (median: 21.77 vs. 18.84 kg/m^2^; *p* = 0.023) compared to subjects with a high miRNA level. Patients with greater FM at week I had significantly lower expression of the studied miRNA compared to males with lower FM values (median: 23.5 vs. 14.26 kg; *p* = 0.027). Moreover, patients with low miR-5682 expression had significantly greater FM measured at the IVth week of the treatment (median: 20.47 vs. 14.02 kg; *p* = 0.011). In addition, male subjects with low expression of miR-5682 had significantly greater FFM values measured both at the IVth week of RT (median: 50.47 vs. 42.51 kg; *p* = 0.011) and after the completion of treatment (VII) (median: 53.41 vs. 44.91 kg; *p* = 0.009), comparing between patients whose miR-5682 expression level was higher. Patients with high expression of the miRNA had higher median FFMI value measuring at the IVth week of RT (17.07 vs. 15.04 kg/m^2^; *p* = 0.006) and after the end of treatment (week VII) (18.56 vs. 15.04 kg/m^2^; *p* = 0.038). LC patients with low miRNA expression also demonstrated greater nFFMI values during the therapy course (week IV) and after the completion of treatment (week VII), in contrast to other patients, whose miR-5682 expression level was high (median: 17.07 vs. 15.04 kg/m^2^; *p* = 0.006 and 19.11 vs. 15.68 kg/m^2^; *p* = 0.005, respectively).

Regarding the laboratory testing, patients with low miR-5682 expression had significantly higher plasma albumin concentration after the VIIth week of treatment, in contrast to the male with high miR-5682 expression (median: 3.39 vs. 2.98 g/dL; *p* = 0.015) and lower plasma CRP concentration before the treatment (median: 3.98 vs. 5.69 mg/L; *p* = 0.047) (Table 3).

Patients with both mild and severe malnutrition, as defined by SGA assessment, showed significantly higher level of miR-5682 expression compared to well-nourished patients (median: 1.55 vs. 0.88; *p* = 0.045). Moreover, male subjects with CWL had significantly higher miR-5682 expression compared to patients with a lack of CWL (median: 1.37 vs. 0.52; *p* = 0.039) (Table 4).

### 3.3. Correlation between miR-5682 Expression and Parameters Reflecting Nutritional Status 

We recorded that miR-5682 expression negatively correlated with BMI at all analyzed measurement points, as follows: I − rho = −0.270; *p* = 0.044, IV − rho = −0.331; *p* = 0.012 and VII − rho = −0.337; *p* = 0.011. A statistically significant negative correlation was also found between the FM after the IVth week of RT and miR-5682 (rho = −0.357; *p* = 0.015) and a negative correlation between FFM and studied miRNA was found after the VIIth week of the treatment (rho = −0.335; *p* = 0.022). A similar significant correlation was observed for nFFMI after the VIIth week of RT (rho = −0.440; *p* = 0.002). Moreover, miR-5682 expression negatively correlated with the both TP and albumin concentration at the VIIth measurement point (rho = −0.333; *p* = 0.018 and rho = −0.442; *p* = 0.001, respectively). There was also a significant positive correlation between SGA scoring and miRNA expression (rho = 0.268; *p* = 0.045) (Table S2).

### 3.4. Diagnostic Usefulness of the Assessment of mi-5682 Expression in Predicting Nutritional Disorders and Its Prognostic Value

**Figure 1:**
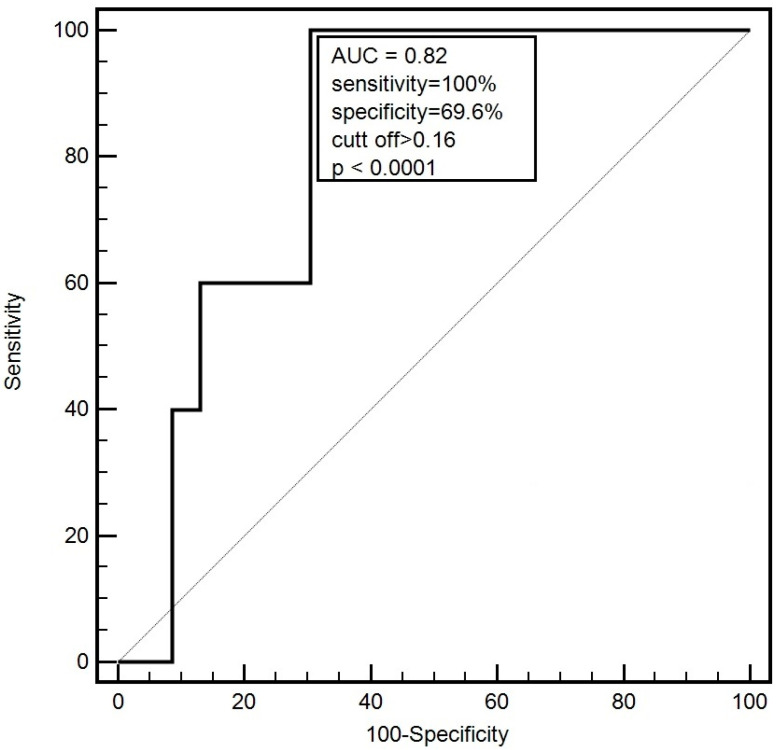
Diagnostic accuracy of miR-5682 conducted by ROC analysis with an AUC calculation. A test for distinguish between patients qualified as severely malnourished (C group according to the SGA scale) with corresponding low plasma albumin concentration and other patients.

Before the commencement of RT (week I), expression level of miR-5682 allowed to distinguish between patients demonstrating simultaneous presence of SGA-C score and plasma albumin concentration <3.2 g/dL and other LC individuals with 100% sensitivity and 69.6% specificity (AUC = 0.820; cut-off > 0.16; *p* < 0.0001) (Figure 1).

**Figure 2:**
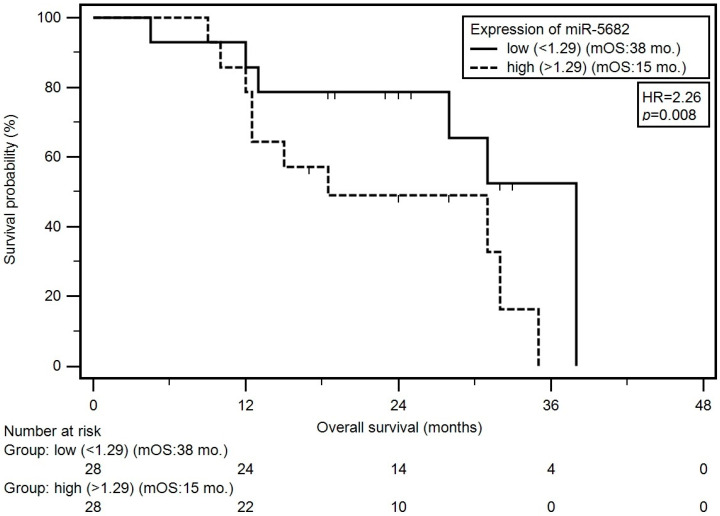
Kaplan–Meier estimator analysis presenting the relationship between miR-5682 expression level and overall survival of LC patients.

The univariate survival analysis (Kaplan–Meier log-rank test) demonstrated that patients with N > 1 category and LC individuals with the M1 category had a significantly higher (approximately 5.5- and 15-fold, respectively) risk of an early death incidence (HR = 5.49; *p* = 0.006 and HR = 14.54; *p* < 0.001, respectively) compared to other patients. Moreover, patients with a high expression level of miR-5682 had significantly shorter median overall survival (OS) in contrast to the individuals demonstrating low miRNA expression (median OS: 38 months vs. 15 months; HR = 2.26; *p* = 0.008) (Figure 2). The multivariate analysis (Cox proportional hazard model) selected N > 1 stage (HR = 19.13; *p* = 0.013), C-RT (HR = 8.65; *p* = 0.016) and a high miR-5682 expression level (HR = 3.82; *p* = 0.012) were unfavorable dependent factors affecting patients’ survival. The results for overall survival analysis including univariate and multivariate model are presented in Table 5. 

## 4. Discussion

Nutritional deficiencies are commonly diagnosed in HNC patients, and they are exposed most frequently in LC individuals [16]. In addition, a specific tumor localization spreading in the area of larynx directly impedes patients’ oral ingestion and alter appetite and taste senses [17]. Moreover, malnutrition is a common problem in HNC patients who undergo radiation therapy [18]. Nutritional deficits negatively affect not only both the patient’s survival and quality of life, but they also lead to a higher risk of manifestation of adverse effects of the treatment [17]. Treatment-related toxicity and the development of tumor-promoting inflammation are believed as the leading mechanisms involved in the development of nutritional deficiencies in RT-treated HNC patients.

Recent studies evidenced that weight loss is a frequent event emerging as a result of RT, and it can be found even in 57% of LC cases [19]. Moreover, pre-treatment BMI values in LC patients were found higher compared to those measured after the completion of RT (23.7 kg/m^2^ vs. <20 kg/m^2^; *p* = 0.05) [10]. In the another study, in 44% of patients with LC weight loss of at least 5% of the initial body mass was reported after completion of RT. In total, 15% of the reported patients lost more than 10% of initial body weight as a consequence of RT [19]. Currently, systemic inflammation is considered as a leading mechanism contributing to development of nutritional disorders in cancer patients [20]. It was also recorded that RT-based therapy and its side-effects can promote inflammatory response by the overproduction of pro-inflammatory agents. Specifically, increased levels of pro-inflammatory cytokines (e.g., IL-6, TNF-α, INF-γ) cause a gradual loss of adipose and muscle tissue. Taking into consideration above-mentioned clinical challenges, the investigation of reliable markers allowing for early detection of malnutrition in RT-treated patients seems to be an urgency. Among the prospective biomarkers reflecting nutritional deficits in cancer patients, miRNAs play a key role in the regulation of genes encoding proteins that control the metabolism of adipocyte and muscle cells. They also regulate inflammatory response; hence, miRNAs are believed as adequate biomarkers reflecting malnutrition in cancer [21]. However, miRNAs have not been widely verified and accepted as clinical diagnostic standards so far. According to the literature data, miR-29a, miR-21 and miR-155, for instance, can play a putative role in the regulation of systemic inflammation accompanying either malnutrition or cachexia [22,23]. Nevertheless, in the routine diagnostics of cancer patients the subjective tools (SGA, NRS questionnaires), anthropometric measurements and laboratory parameters (albumin, CRP) are constantly used.

It should be noted that in the available literature data, there is a lack of studies evaluating the relationship between miR-5682 expression and nutritional status of cancer patients. We have only found that miR-5682 plays an important role in the regulation of inflammatory responses via modulation of the interleukin 17 (IL-17) pathway [24]. Furthermore, it has been confirmed that IL-17 activates the JAK/STAT3 signaling pathway during progression of muscle atrophy, and its high level also promotes inflammation in visceral adipose tissue (VAT) and initiates lipolysis [25,26]. These observations seem to partly meet results of bioinformatics findings.

Regarding the identified miRNAs as putative biomarkers of malnutrition, miR-130a was examined in plasma samples of 70 patients with HNC, including 38 individuals (54.3%) with tumor of the larynx. Patients with a decreased expression of miR-130a demonstrated an increased body mass before RT compared to individuals with higher expression of this miRNA (68.3 vs. 60.5 kg; *p* = 0.036). Additionally, a lower level of miR-130a expression was related to a greater loss of body weight during treatment course [27]. In another study, lower expression of miR-511-3p was observed in severely malnourished (according to SGA scale) HNC patients (0.93 vs. 6.27; *p* = 0.0001). Additionally, this miRNA significantly correlated with BMI (*p* = 0.013), albumin (*p* = 0.009), CRP (*p* = 0.004), FFM (*p* = 0.015), FFMI (*p* = 0.049) and NRI (*p* = 0.0004) in HNC patients undergoing RT [28]. We observed higher miR-5682 expression in LC subjects with severe malnutrition (SGA-C) compared to mildly malnourished and well-nourished ones (SGA-A+B) (1.55 vs. 0.88; *p* = 0.045). In the study conducted on a group of 56 men with HNC, the expression of miR-181a in plasma was analyzed. Patients with high miR-181a level were characterized by lower FM (median: 39.15 vs. 47.01 kg; *p* = 0.003) and FFM (median: 14.03 vs. 45.99 kg; *p* = 0.01) after the seventh week of RT [29]. In our study, parameters obtained from BIA (FM, FFM, FFM, nFFMI) were recorded significantly reduced at different measurement points in patients demonstrating higher expression level of miR-5682. The occurrence of nutritional deficiencies in HNC patients was associated with the low blood concentration of albumin and CRP [30]. We noticed a lower albumin concentration in patients with higher miR-5682 expression measured after completion of the treatment. Additionally, we recorded significant correlation between miR-5682 expression and BMI, FM, FFM, nFFMI, TP and albumin. Analysis of miRNAs can be used as a valuable tool for distinguish between well-nourished patients and individuals suffering from nutritional deficiencies [27,28]. ROC analysis for miR-5682 allowed us to distinguish between SGA-C patients with a corresponding presence of low plasma albumin level (<3.2 g/dL) and other individuals with sensitivity of 100% and specificity of 69.6% (AUC = 0.820). Focusing on a prognostic value of different miRNAs in HNC, patients with lower expression of miR-130a had significantly shorter OS in contrast to those with higher miRNA expression (median OS: 32.5 vs. 38 months; HR = 2.582; *p* = 0.087) [27]. Zhang et al. reported that higher expression of miR-23a was significantly related to shorter 5 year OS compared to LC patients demonstrating lower expression of this miRNA (HR = 7.41; *p* < 0.001) [31]. Additionally, high expression of miR-149 was found to be associated with shorter OS in LC patients [32]. Ding et al. also noted that LC patients with high expression ofmiR-195 had longer OS compared to the other patients (median OS: 57 vs. 39 moths; *p* = 0.015) [33]. We observed that males with high expression level of miR-5682 had significantly shorted OS compared to individuals with low expression (median OS: 15 vs. 38 months; HR = 2.26; *p* = 0.008).

## 5. Conclusions

Our study includes some limitations: the retrospective manner of the analysis, a small sample size, and only male patients were recruited to study. The patients included in the study are quite homogeneous in tumor stage and type of the treatment. Moreover, we use only CRP plasma concentrations in order to assess the inflammation response. Nevertheless, an analysis of miR-5682 expression demonstrates a potential clinical utility in selection of LC patients suffering from nutritional deficiencies developing as a consequence of RT-based therapy. 

## Figures and Tables

**Table 1:** Detailed characteristics of the studied group.

Factor		*n* = 56 (%)
Age [years]	Median (range) >65 ≤65	65 (42–87) 42 (75%) 14 (25%)
T stage	T1 T2 T3 T4	2 (3.6%) 12 (21.4%) 20 (35.7%) 22 (39.3%)
N stage	N0 N1 N2 N3	28 (50%) 10 (17.9%) 16 (28.6%) 2 (3.6%)
M stage	Mx M0 M1	2 (3.6%) 52 (92.9%) 2 (3.6%)
Disease stage (TNM)	III IVA IVB	26 (46.4%) 26 (46.4%) 4 (7.1%)
Performance status (PS)	≤1 >1	52 (92.9%) 4 (7.1%)
Type of treatment	Surgery + RT Surgery + C-RT	33 (58.9%) 23 (41.1%)
Alcohol consumption	Yes No	32 (57.1%) 24 (42.9%)
Smoking status	Smoker Non-smoker	42 (75%) 14 (25%)
	Current smoker Former smoker	40 (95.2%) 2 (4.8%)
Relative expression of miRNA-5682	Mean ± SD median (range)	2.95 ± 1.56 1.29 (0.01–18.19)

Abbreviations: C-RT—chemoradiotherapy; ECOG—Eastern Cooperative Oncology Group; M—metastatic spread; N—lymph node involvement; RT—radiotherapy; SD—standard deviation; T—tumor site and size; TNM tumor, node, and metastasis staging.

**Table 2:** Baseline nutritional characteristics of the male LC patients.

Factor		*n* = 56 (%)
Weight [kg]	Mean ± SD, median (range)	64.73 ± 11.83 64.75 (43–91)
Body mass index (BMI) [kg/m^2^]	Mean± SD median (range) ≥18.5 <18.5	22.99 ± 4.32 18.5 (14.53–34.37) 44 (78.6%) 12 (21.4%)
Subjective Global Assessment (SGA)	A B C	8 (14.3%) 22 (39.3%) 26 (46.4%)
Nutritional Risk Score (NRS-2002)	2 3 4 5	40 (71.4%) 12 (21.4%) 2 (3.6%) 2 (3.6%)
Critical weight loss (CWL)	Yes No	22 (39.3%) 34 (60.7%)
Nutritional Risk Index (NRI)	Mild Moderate Severe	8 (14.3%) 42 (75%) 6 (10.7%)
FM [kg]	Mean ± SD median (range)	18.59 ± 8.05 18.48 (7.27–34.25)
FFM [kg]	Mean ± SD median (range)	48.21 ± 6.72 46.79 (30.31–60.64)
FFMI [kg/m^2^]	Mean ± SD median (range)	16.85 ± 2.13 16.59 (11.55–20.99)
nFFMI [kg/m^2^]	Mean ± SD median (range)	17.48 ± 2.23 17.44 (12.65–22.21)
CRP [mg/L]	Mean ± SD, median (range)	7.45 ± 8.67 4.92 (0.14–35)
TP [g/L]	Mean ± SD median (range)	6.62 ± 0.53 6.59 (5.52–7.52)
Albumin [g/dL]	Mean ± SD median (range)	3.35 ± 0.26 3.30 (2.81–3.92)
Prealbumin [g/dL]	Mean ± SD median (range)	0.23 ± 0.08 0.20 (0.1–0.4)
Transferrin [g/L]	Mean ± SD median (range)	2.33 ± 0.54 2.40 (1.2–3.2)

Abbreviations: BMI—body mass index; CRP—C-reactive protein; C-RT—chemoradiotherapy; FFM—fat-free mass; FFMI—fat-free mass index; FM—fat mass; nFFMI—normalized fat-free mass index; CWL—critical weight loss; ECOG—Eastern Cooperative Oncology Group; M—metastatic spread; N—lymph node involvement; NRI—nutrition risk index; NRS-2002—Nutritional Risk Screening 2002; RT—radiotherapy; SD—standard deviation; SGA—subjective global assessment; T—tumor site and size; TNM tumor, node, and metastasis staging; TP—total protein.

**Table 3:** Differences in nutritional status between patients with high and low expression of miR-5682 measured at the different points of RT-based therapy (weeks: I, IV and VII).

Factor	Measurement (Week)	miR-5682 Expression		*p*
		High Median (IQR) (*n* = 28)	Low Median (IQR) (*n* = 28)	
Weight [kg]	I	62 (54–72)	68 (59.5–71.5)	0.455
	IV	60.5 (52–68)	65 (55.5–67.75)	0.323
	VII	56 (51–66)	62 (54.25–68)	0.285
Weight loss during treatment [%]	I–IV	4.58 (1.49–10.52)	3.38 (1.92–8.69)	0.947
	IV–VII	3.84 (2–7.69)	3.92 (1.59–9.37)	0.818
	I–VII	6.27 (3.70–13.64)	4.35 (2.08–11.94)	0.512
BMI [kg/m^2^]	I	21.96 (17.96–23.46)	23.78 (22.36–26.51)	0.018 *
	IV	19.82 (18.31–22.98)	22.67 (20.89–23.53)	0.009 *
	VII	18.84 (16.79–23.11)	21.77 (19.56–24.09)	0.023 *
FM [kg]	I	14.26 (9.63–22.29)	23.50 (18.35–31.30)	0.027 *
	IV	14.02 (10.74–22.59)	20.47 (13.35–23.82)	0.011 *
	VII	13.46 (12.56–20.78)	14.81 (11.37–18.49)	0.857
FM [%]	I	24.76 (21.97–29.36)	30.07 (24.12–34.61)	0.101
	IV	24.46 (20.73–35.04)	32.63 (23.55–37.27)	0.344
	VII	25.08 (22.77–28.54)	24.29 (18.91–25.61)	0.071
FFM [kg]	I	46.60 (42.71–53.47)	47.57 (44.98–53.7)	0.212
	IV	42.51 (39.75–46.29)	50.47 (42.78–52.03)	0.011 *
	VII	44.91 (38.57–47.79)	53.41 (44.44–55.36)	0.009 *
FFM [%]	I	75.19 (70.66–82.13)	69.80 (65.15–75.88)	0.076
	IV	75.71 (64.56–79.73)	67.65 (64.93–75.11)	0.149
	VII	75.04 (71.05–77.82)	75.68 (74.64–80.29)	0.071
FFMI [kg/m^2^]	I	16.59 (15.98–17.32)	16.64 (15.37–18.57)	0.533
	IV	15.04 (13.67–16.21)	17.07 (15.04–18.69)	0.006 *
	VII	15.04 (14.28–16.62)	18.56 (16.5–18.92)	0.038 *
nFFMI [kg/m^2^]	I	17.20 (16.57–18.46)	17.61 (15.93–18.81)	0.446
	IV	15.6 (14.22–16.88)	17.50 (15.68–19.54)	0.019 *
	VII	15.68 (13.91–16.50)	19.11 (17.36–19.65)	0.005 *
CRP [mg/L]	I	5.69 (1.55–7.22)	3.98 (1.25–11.86)	0.047 *
TP [g/L]	I	6.63 (6.30–7.10)	6.59 (6.10–6.84)	0.309
	IV	6.61 (6.18–7.24)	6.51 (6.42–6.82)	0.667
	VII	6.17 (5.58–7.04)	6.67 (6.58–6.77)	0.085
Albumin [g/dL]	I	3.22 (3.11–3.3)	3.37 (3.26–3.45)	0.094
	IV	3.13 (2.94–3.49)	3.4 (3.22–3.46)	0.183
	VII	2.98 (2.8–3.25)	3.39 (3.07–3.5)	0.015 *

*—statistically significant results. Abbreviations: BMI—body mass index; CRP—C reactive protein; FFM—fat-free mass; FFMI—fat-free mass index; FM—fat mass; nFFMI—normalized fat-free mass index; IQR—interquartile range; TP—total protein.

**Table 4:** Comparisons of the relative expression of miR-5682 depending on values of parameters reflecting patients’ nutritional status.

Nutritional Parameters		Comparisons	
		Median (IQR)	*p*
SGA	A	1.06 (0.41–2.35)	0.302
	B or C	1.32 (0.32–4.32)	
	A or B	0.88 (0.32–2.34)	0.045 *
	C	1.55 (0.52–4.41)	
SGA	A	1.06 (0.41–2.35)	0.128 ^#^
	B	0.88 (0.22–2.34)	
	C	1.55 (0.52–4.41)	
NRS-2002	<3	1.38 (0.6–3.82)	0.309
	>3	0.91 (0.21–2.89)	
CWL	Yes	1.37 (0.88–3.39)	0.039 *
	No	0.52 (0.20–4.24)	
CRP [mg/L]	>5	1.34 (0.81–2.74)	0.238
	<5	0.85 (0.2–4.24)	
CRP [mg/L]	>10	1.27 (0.52–1.37)	0.472
	<10	1.49 (0.24–4.24)	
Weight loss 5%	Yes	1.32 (0.81–4.67)	0.373
	No	1.10 (0.24–2.74)	
Weight loss 10%	Yes	0.81 (0.22–4.99)	0.791
	No	1.31 (0.39–3.39)	

*—statistically significant results; #—result calculated by Kruskal–Wallis test. Abbreviations: CRP—C reactive protein; CWL—Critical Weight Loss; IQR—interquartile range; NRS-2002—Nutritional Risk Screening 2002; SGA—Patient-Generated Subjective Global Assessment.

**Table 5:** Significant parameters affecting OS of LC patients selected by uni- and multivariate analysis.

Factor	Univariate Analysis		Multivariate Analysis	
	HR [95%CI]	*p*	HR [95%CI]	*p*
Smoking history (yes)	-	NS		
N stage (N1-3)	5.49 [0.23–133.10]	0.006*	19.13 [1.76–196.57]	0.013 *
M stage (M1)	14.54 [0.08–2505.86]	<0.0001 *	-	NS
Treatment (concurrent C-RT)	-	NS	8.65 [1.52–49.37]	0.016 *
Relative expression of miR-5682 (high)	2.26 [1.14–4.52]	0.008 *	3.82 [1.33–10.93]	0.012 *

*—statistically significant results. Abbreviations: CI—confidence interval; C-RT—chemoradiotherapy; HR—hazard ratio; M—metastatic spread; N—lymph node involvement; NS—non-significant.

## Data Availability

The data presented in this study are available in this article and Supplementary Materials.

## Supplementary Materials

The following supporting information can be downloaded at: https://www.mdpi.com/article/10.3390/genes15050556/s1, Table S1: Differences in miR-5682 expression among patients with different clinical-demographic features; Table S2: Correlation between demographic, clinical and nutritional variables and the relative expression of miR-5682; Figure S1: Ct values of internal control (miR-26-3p) and miR-5682 in studied patients.
