# Supplementary material for: The Relationship between miR-5682 and Nutritional Status of Radiotherapy-Treated Male Laryngeal Cancer Patients
Source: Genes (Basel). 2024 Apr 27;15(5):556. doi: 10.3390/genes15050556 (PMC11120884; doi:10.3390/genes15050556)
Supplement: Supplementary file 1 [file genes-15-00556-s001.zip › genes-2964538-supplementary.pdf]

**Table S1.** Differences in miR-5682 expression among patients with different clinical-demographic features

| Factor | | Relative expression of miRNA | |
|---|---|---|---|
| | | Median (IQR) | *p* |
| Age (years) | > 65<br>≤ 65 | 1.37 (0.80-4.24)<br>0.39 (0.06-1.49) | 0.576 |
| Disease stage (TNM) | III<br>IVA-IVC | 1.27 (022-2.34)<br>1.49 (0.52-4.67) | 0.081 |
| T stage | T1-3<br>T4 | 1.27 (0.24-2.74)<br>1.49 (0.39-4.41) | 0.568 |
| N stage | N0<br>N1-3 | 1.27 (0.24-3.39)<br>4.41 (-) | 0.133 |
| M stage | M0<br>M1 | 1.27 (0.24-3.39)<br>4.24 (-) | 0.185 |
| Performance status | ≤1<br>>1 | 1.08 (0.24-2.74)<br>5.94 (4.24-7.65) | 0.015* |
| Alcohol consumption | Yes<br>No | 1.46 (0.45-3.49)<br>1.08 (0.14-4.03) | 0.466 |
| Smoking status | Smoker<br>Non-smoker | 1.37 (0.52-3.39)<br>0.80 (0.03-4.24) | 0.120 |

* - statistically significant results

Abbreviations: IQR - interquartile range; M - metastatic spread; N - lymph node involvement; T - tumor site and size.

**Table S2.** Correlation between demographic, clinical and nutritional variables and the relative expression of miR-5682

| Factor | miR-5682 | |
|---|---|---|
| | Univariable | |
| | rho | *p* |
| Age [years] | 0.374 | 0.379 |
| T stage | 0.078 | 0.566 |
| N stage | 0.137 | 0.314 |
| M stage | 0.133 | 0.328 |
| Diseasestage (TNM) | 0.235 | 0.081 |
| Weight I [kg] | -0.147 | 0.278 |
| WeightIV[kg] | -0.224 | 0.096 |
| WeightVII[kg] | -0.254 | 0.058 |
| BMI I [kg/m$^2$] | -0.270 | 0.044* |
| BMI IV [kg/m$^2$] | -0.331 | 0.012* |
| BMI VII [kg/m$^2$] | -0.337 | 0.011* |
| FM I [kg] | -0.201 | 0.136 |
| FM IV [kg] | -0.357 | 0.015* |
| FM VII [kg] | -0.058 | 0.700 |
| FM I [%] | -0.197 | 0.145 |
| FM IV [%] | -0.241 | 0.106 |
| FM VII [%] | 0.147 | 0.328 |
| FFM I [kg] | -0.061 | 0.656 |
| FFM IV [kg] | -0.185 | 0.218 |
| FFM VII [kg] | -0.335 | 0.022* |
| FFMI [%] | 0.208 | 0.124 |
| FFMIV [%] | 0.235 | 0.115 |
| FFMVII [%] | -0.140 | 0.352 |
| FFMI I [kg/m$^2$] | 0.051 | 0.715 |
| FFMI IV [kg/m$^2$] | -0.289 | 0.051 |
| FFMI VII [kg/m$^2$] | -0.183 | 0.222 |
| nFFMI I [kg/m$^2$] | -0.008 | 0.951 |
| nFFMI IV [kg/m$^2$] | -0.258 | 0.083 |
| nFFMI VII [kg/m$^2$] | -0.440 | 0.002* |
| TP I [g/L] | 0.042 | 0.759 |
| TP IV [g/L] | -0.132 | 0.359 |
| TP VII [g/L] | -0.333 | 0.018* |

| | | |
|---|---|---|
| Albumin I [g/dL] | -0.153 | 0.261 |
| Albumin IV [g/dL] | -0.140 | 0.330 |
| Albumin VII [g/dL] | -0.442 | 0.001* |
| Prealbumin [mmol/L] | -0.130 | 0.340 |
| Transferrin [g/L] | -0.053 | 0.699 |
| CRP [mg/L] | 0.005 | 0.969 |
| NRI | -0.073 | 0.591 |
| Duration of nutritional support[days] | -0.500 | 0.312 |
| NRS-2002 | -0.139 | 0.305 |
| SGA | 0.268 | 0.045* |

*—statistically significant results

Abbreviations: BMI—body mass index; CRP—C-reactive protein; FFM—fat free mass; FFMI—fat-free mass index; FM—fat mass; M—metastatic spread; N—lymph node involvement; nFFMI—normalized fat-free mass index; NRI—nutrition risk index; NRS-2002—Nutritional Risk Screening 2002; SGA—subjective global assessment; T—tumor site and size and TNM—tumor, node, and metastasis staging.

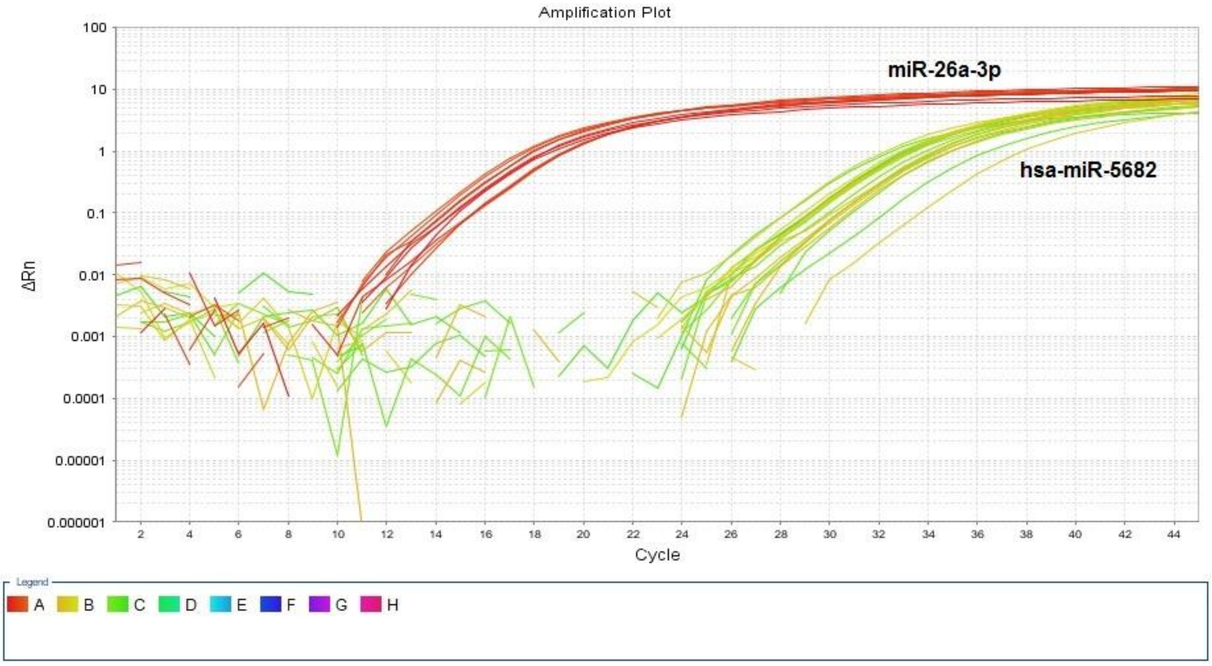


**Figure S1.** Ct values of internal control (miR-26-3p) and miR-5682 in studied patients.
